# Practice and community nurses' views and experiences of helping people manage risk factors for recurrent lower limb cellulitis: A qualitative interview study

**DOI:** 10.1002/ski2.395

**Published:** 2024-04-30

**Authors:** Ingrid Muller, Emma Teasdale, Fiona Cowdell, Peter Smart, Miriam Santer, Nick Francis

**Affiliations:** ^1^ Primary Care Research Centre Faculty of Medicine University of Southampton Southampton UK; ^2^ Faculty of Health, Education and Life Sciences Birmingham City University Birmingham UK; ^3^ Patient Representative Centre for Evidence Based Dermatology University of Nottingham Nottingham UK

## Abstract

**Background:**

Cellulitis is a painful infection of the skin and underlying tissues, commonly affecting the lower leg. Approximately one‐third of people experience recurrence. Nurses who work in general practice (practice nurses) and see people at home (community or district nurses) could have an important role in managing risk factors for cellulitis, such as long‐term leg swelling, wound care and skin care.

**Objective:**

To explore practice and community nurses' views and experiences of helping people to manage risk factors for recurrent lower limb cellulitis.

**Methods:**

Semi‐structured, telephone interviews with 21 practice and community nurses in England from October 2020 to March 2021. Interviews were transcribed verbatim and analysed using reflexive thematic analysis.

**Results:**

Nurses face multiple challenges when supporting people to manage risk factors for recurrent lower limb cellulitis. Key challenges include limited time and access to resources such as Doppler equipment, and the physical and psychosocial capabilities of patients to self‐manage. Nurses identified potential strategies to overcome these challenges, such as placing greater emphasis on prevention and supporting self‐management by providing resources for patients and support networks (paid and unpaid carers) to reinforce knowledge post‐consultation and develop skills to self‐care.

**Conclusions:**

We identified a need to develop and evaluate resources, such as support materials, for nurses to use to help patients reduce their risk of recurrent cellulitis.

## Graphical abstract

This study aimed to explore practice and community nurse's experiences of supporting people with recurrent lower leg cellulitis. Interviews with 21 nurses identified a range of challenges, and potential solutions to these, they face when supporting people to manage risk factors for recurrent lower limb cellulitis.

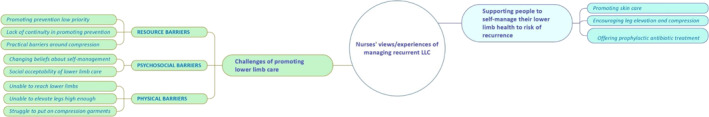

**What is already known about this topic?**

- Cellulitis is a common skin infection that often affects the lower legs. It can be painful, serious and can cause long‐term complications.
- Approximately a third of people with leg cellulitis have recurrent episodes, which can lead to frequent use of antibiotics.
- Practice and community nurses could have an important role in managing risk factors for recurrent lower limb cellulitis.

**What does this study add?**

- Nurses said that supporting patients manage risk factors for lower limb cellulitis was a core part of their role, but described multiple challenges, specifically lack of resources and patients' differing physical and psychosocial abilities.

**What are the clinical implications of the work?**

- Cellulitis management could be more proactive, with a greater emphasis on prevention.
- There is a need to develop and evaluate patient materials and support tools to help people with cellulitis reduce their risk of recurrence.

## 1 INTRODUCTION

Cellulitis is a painful and potentially serious bacterial skin infection that is characterised by inflammation (redness or darkening of skin depending on skin tone, swelling, warmth and tenderness) of the affected area.^1^, ^2^ It affects approximately 1 in 40 people per year,^3^, ^4^ and most often affects the lower legs.^5^ Cellulitis is associated with significant morbidity^6^, ^7^ and substantial healthcare burden, both in terms of costs and resources.^8^, ^9^ It impacts on quality of life due to severe pain and inflammation, often accompanied by a high fever, nausea, rigours and feeling unwell.^2^, ^6^, ^10^ Cellulitis can also lead to long‐term complications such as lymphoedema and decreased mobility.^3^ It is a common reason for general practice (GP) consultations, and resulted in over 105 000 hospital admissions in England during 2019–2020.^11^ Cellulitis is also a common reason to be prescribed antibiotics^12^ and use of antibiotics is the main driver of antimicrobial resistance, one of the most pressing public health threats of our time.^13^

Approximately one third of people with cellulitis have repeat episodes.^3^ Each episode of lower limb cellulitis increases the likelihood of future episodes, leading to a cycle of recurrent disease.^14^ Risk factors for recurrence include previous episode of cellulitis; obesity; diabetes and breaks in the skin.^14^, ^15^ Leg oedema, tinea pedis and varicose eczema are important risk factors for lower limb cellulitis as bacteria can enter through areas of dry, cracked skin.^16^ The skin barrier can also be disrupted by pressure ulcers and venous leg ulcers. Older people and people with chronic venous insufficiency are at greater risk of developing lower limb cellulitis.^5^, ^17^, ^18^ Recurrent cellulitis can lead to chronic dermal changes and damage to the lymphatic system that can result in lymphoedema, which further increases the risk of subsequent episodes of cellulitis.^7^, ^17^, ^18^

The only intervention with good trial evidence for preventing recurrent disease is long‐term, low‐dose antibiotics,^3^, ^19^, ^20^ which can cause side effects and promote the development of antibiotic resistance. Current research suggests that prophylactic antibiotic treatment may need to be used indefinitely to reduce the risk of future episodes and may be less effective in people with a BMI > 33 and pre‐existing leg oedema.^21^

In a James Lind Alliance research priority setting exercise identifying non‐antibiotic approaches to reducing the risk of recurrent cellulitis was amongst the top 10 priorities.^22^ There is limited evidence about optimal strategies to reduce risk of cellulitis recurrence. However, clinical consensus supports approaches to: measures to maintain skin integrity (e.g. checking for and treating skin conditions such as tinea pedis and varicose eczema); hygiene and emollient therapy (washing and moisturising the skin); and strategies to reduce leg swelling (use of compression garments, exercise and leg elevation).^3^, ^5^, ^15^, ^16^, ^21^, ^23^, ^24^

District nurses, community nurses and GP practice nurses play a key role in supporting patients to avoid recurrent lower limb cellulitis. Nurses commonly manage chronic venous stasis and lymphoedema, measurement for compression stockings or wraps, management of ulcers and wounds, skin care, and may be involved in testing for fungal infections. District and community nurses typically see housebound and care home‐based patients, in whom recurrent lower leg oedema and cellulitis are common. Therefore, nurses are an important resource for understanding some of the key challenges in reducing the risk of recurrence of cellulitis in this population and could potentially deliver interventions or support to help people reduce risk of recurrence. Early and accurate assessment of worsening leg symptoms is essential for improving outcomes and avoiding unnecessary antibiotic use for these patients, and nursing teams play a key role in this as well. However, there is lack of evidence about the views and experiences of nurses working in primary care on reducing the risk of recurrent lower limb cellulitis.

The aim of this study was to explore practice and community nurses' views and experiences of helping people manage risk factors for recurrent lower limb cellulitis.

## 2 METHODS

### 2.1 Study design

Qualitative study comprising semi‐structured interviews with registered nurses working in GP practices (practice nurses) and community nursing teams (community and district nurses). We obtained ethics approval from the Faculty of Medicine Research Ethics Committee, University of Southampton (Ref: 60299).

### 2.2 Setting and participants

Registered nurses were recruited via research and professional networks in the South of England. Considering the challenges of recruiting during the Covid‐19 pandemic we interviewed a convenience sample at mutually convenient times. We placed no restrictions on age, gender, years of experience, or workplace settings but health care support workers and nursing assistants were excluded.

### 2.3 Data collection

One author (ET), an experienced qualitative researcher, conducted interviews from October 2020 to March 2021. Interviews were conducted via telephone (*n* = 20) or MS Teams (*n* = 1) and lasted an average of 36 min (range 25–55 min). We sought written informed consent prior to conducting the interview. Participants were offered a £15 gift voucher for their time. Interviews followed a semi‐structured topic guide (Box 1) based on existing literature, research team expertise and input from patient/public collaborators. Interviews were audio‐recorded, anonymised and transcribed verbatim.

**BOX 1 Interview topic guide**

1

1. Can you tell me about your experience of managing patients who at risk of having repeat episodes of lower limb cellulitis?
2. Which patients do you think are most at risk of having repeat episodes of lower limb cellulitis?
3. Can you tell me about the ways you help patients reduce the risk of cellulitis coming back again?
4. What are the benefits/challenges? What do you think works well/less well?
5. What do you think could be done differently?
6. What do you think are the most feasible/effective things patients can do to reduce the risk of recurrence?
7. Is there anything that you currently do to support patients with this?
8. What do you think about the following things that patients could do to help prevent lower limb cellulitis from coming back again?
  - Skin care (hygiene and emollient therapy).
  - Recognising and treating fungal foot disease.
  - Exercise and leg elevation.
  - Using compression garments.
  - Recognising and treating eczema and other skin conditions.
9. What would help you to support patients with carrying out these things?
10. How would you feel about having a tool to help support patients to reduce their risk of recurrence?
11. What do you think would make it difficult to deliver such a support tool through primary care or community settings?
12. What do you think would make it easy to deliver such a support tool through primary care or community settings?
13. What do you think would be a suitable format for such a support tool?
14. We are considering developing a website, booklets, or videos:
  - What do you think would be good/bad about a web‐based support tool?
  - What do you think would be good/bad about a patient booklet?
  - What do you think would be good bad about videos?

### 2.4 Data analysis

Data were analysed using reflexive thematic analysis^25^, ^26^ to identify and report patterns across the data. Our process was to (1) familiarise with data, (2) code interview text line by line, (3) develop an initial coding list, (4) explore negative cases to ensure all data considered and (5) produce a detailed coding manual to ensure transparent and systematic coding and to use as a basis for organising initial codes into a hierarchy of broader categories or themes. We augmented this analysis method with coding techniques from grounded theory for example, open coding, line‐by‐line coding, constant comparison.^27^

Codes were derived inductively from the data and grouped together to produce an initial coding frame. Initial coding, identification of descriptive themes and subthemes was carried out by one author (ET) then discussed and iteratively developed with the research team (IM, MS, NF, FC, PS) to offer diverse interpretation of the data and facilitate the generation of analytical themes. We used constant comparison to allow themes from preliminary analyses to inform subsequent interviews. A reflexive approach was adopted taking into consideration the interviewer's positioning as female research psychologist (ET), GP perspectives (MS, NF), nursing perspective (FC) and patient (PS) perspective within the researcher team.

## 3 FINDINGS

### 3.1 Participant characteristics

We conducted 21 semi‐structured interviews between October 2020 and March 2021. We interviewed 13 practice nurses and 8 community nurses working in patients' homes, with a median of 16 years nursing experience **(range 5–42 years).** Participants were predominantly female (*n* = 19) (Table 1).

**TABLE 1:** Participant characteristics.

Characteristics	*N* = 21
Nursing role	
Practice nurse	13
Community nurse	8
Gender	
Female	19
Male	2

### 3.2 Key themes

**FIGURE 1:**
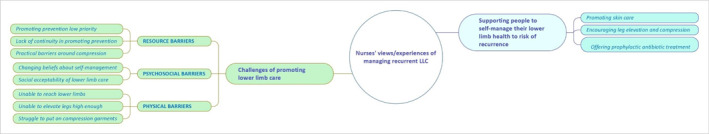
Diagram of key themes.

Two major themes were identified: ***Supporting people to self‐manage their lower limb health to reduce risk of recurrence*** and ***Challenges and suggestions for promoting lower limb care*** (Figure 1). We explore each of the themes and subthemes in detail below and present selected quotes to illustrate each theme.

### 3.3 Supporting people to self‐manage their lower limb health to reduce risk of recurrence

In accordance with National Institute for Health and Care Excellence (NICE) guidelines on managing acute cellulitis in primary care (Box 2), participants described how their predominant focus was on managing the acute episode of lower limb cellulitis by treating the infection with antibiotics and administering leg care restore lower limb health. Focus then shifts to managing underlying/predisposing risk factors or co‐morbidities and advising people about preventative measures to reduce their risk of recurrence. Preventative measures involved promoting self‐management of lower limb health (skin care, encouraging leg elevation and use of compression garments where appropriate) and prophylactic antibiotics in a minority of cases.

**BOX 2 Standard care of lower limb cellulitis (add ref)**

1 *Summary of NICE guidelines on managing acute cellulitis in primary care*

For people with Class I cellulitis (no signs of systemic toxicity and no uncontrolled comorbidities):

- Prescribe a high‐dose oral antibiotic treatment.
  ○ Before treatment, draw around the extent of the infection with a permanent marker pen for future comparison and to track the spread of infection.
- Advise the person to:
  ○ Take paracetamol or ibuprofen for pain and fever. For detailed information on prescribing these analgesics.
  ○ Drink adequate fluids.
  ○ Seek immediate medical advice if antibiotics are not tolerated, the cellulitis becomes worse or if systemic symptoms develop or worsen.
  ○ Elevate the leg for comfort and to relieve oedema (where applicable).
  ○ Avoid the use of compression garments during acute cellulitis.
- Manage any underlying risk factors for cellulitis.
  ○ Manage breaks in the skin, for example, due to eczema, tinea pedis, or leg ulcers, which may become a portal of entry for organisms.
  ○ Manage venous insufficiency.
  ○ Consider referring people with lymphoedema to a specialist clinic.
  ○ Liaise with a district nurse if there is skin blistering, broken skin, exudate, or venous ulceration.
- Identify and manage comorbidities (such as diabetes mellitus) that may cause the cellulitis to spread rapidly, or delay healing.
- Advise on preventative measures to reduce the risk of recurrence, including:
  ○ Weight management if the person is obese.
  ○ The use of emollients to prevent dry skin and cracking.
- Provide patient information on cellulitis. For example:
  ○ Cellulitis or erysipelas published by the British Association of Dermatologists (BAD, www.bad.org.uk).
  ○ About cellulitis published by the Lymphoedema Support Network (www.lymphoedema.org).
  ○ Cellulitis published by National Health Service (NHS) (www.nhs.uk).
- Review the person after 2–3 days depending on clinical judgement, or if local symptoms deteriorate (such as redness or swelling beyond the initial presentation), have severe pain, or they develop systemic symptoms.
- If a person has recurrent episodes of cellulitis (more than two episodes at the same site within 1 year), consider routine referral to secondary care for advice on the use of prophylactic antibiotics.

### 3.4 Promoting skin care

Providing information and advice on skin care was routine for GP and community nurses, and a core part of their role. This included advising people to wash their legs and regularly apply moisturising creams as well as providing suitable moisturising creams (e.g. emollients) and/or advice about suitable moisturising creams to help people manage breaks in their skin (maintain skin integrity).

We do a full skin assessment of everybody that we see, so we would be advising and requesting creams for everybody because well leg care is part of our job. That would just be our general thing to do, regardless of whether they've had cellulitis or not… It'd be normal practice to promote well leg care for all of our patients.

P19 (community nurse, >10 years exp)

A common view expressed was that ‘decent skin care’ was the most feasible self‐management strategy that people could adopt to try to reduce their risk of recurrence if they were physically able to self‐care.

I think it's probably just the emollients. I'd probably say it's the lowest barrier, the easiest thing to do.

P9 (practice nurse, 5–10 years exp)

In contrast, identifying and managing skin conditions that could cause breaks in the skin barrier, such as fungal foot infections, was not viewed as part of their remit. While some practice nurses did mention treating fungal foot infections as a potential preventative measure, they expressed a lack of confidence in managing such infections and indicated that they would tend to refer to podiatry services or GP management.

I don't know if we are particularly good at spotting fungal issues, but then it would be you have request something from the GP, or I think if it's on the feet we try to be guided by the podiatrists. I think people can be reluctant to make decisions about foot‐related things until they've had podiatry input.

P20 (community nurse, 5–10 years exp)

### 3.5 Encouraging leg elevation and use of compression garments

Compression therapy and leg elevation were widely viewed as effective strategies to reduce leg swelling and therefore were also commonly promoted to people as a way in which they could reduce their risk of recurrent lower limb cellulitis.

If we can get them to see the benefits of compression and they actually will wear it, then it works wonders. Once their legs are down and the skin is in good condition and there's no ulcers or skin breaks, then we get them into hosiery as soon as we can, so they can self‐manage.

P7 (practice nurse, >10 years exp)

I say to anyone who's got lower leg problems, you need to elevate your leg, even if it's just an hour in the afternoon with your feet up on a stool or going back to bed for an hour in the afternoon. It's one of those things where we're constantly prompting and nagging.

P20 (community nurse, 5–10 years exp)

### 3.6 Offering long‐term prophylactic antibiotic treatment

Offering long‐term prophylactic antibiotic treatment was commonly viewed as effective in reducing the risk of recurrence but only as a last resort and in severe cases. Some participants reported experiences of promoting long‐term antibiotics but only in cases of persistent recurrence that is, multiple recurrences per year.

We do not want to advocate long‐term use of antibiotics, but it's very effective… That's not suitable for the wider range, but I think it's just important to remember that people who are having regular bouts…between four to six times a year should be explored for long‐term antibiotic use…. it's not to be forgotten. It is within the arsenal, even though it should be a last resort.

P9 (practice nurse, 5–10 years exp)

### 3.7 Challenges and suggestions for promoting lower limb care

Nurses universally recognised the need for preventative measures, but many experienced challenges and felt frustrated by the cycle of recurrence for some people. Common challenges reported by participants related to resource barriers, physical barriers, and psychosocial barriers around self‐managing lower limb health.

## 4 RESOURCE BARRIERS

### 4.1 Promoting prevention viewed as low priority

Nurses reported focusing primarily on treating the infection, wound healing, and skin care to restore lower limb health. Promoting self‐management was challenging due to due to time constraints and nurses recognised this could lead to a cycle of recurrence for some people.

‘Because there's no longer a visible wound, there's a bit of complacency, I think, around: “Oh, that's job done now.” I'd like to think, at least people get measured for some stockings; but actually, a lot of the time, that's as much as, maybe, they do, before they get discharged from our service ‐ and actually, they're back on the books within a month… there hasn't been any chat around why wearing these stockings for the rest of your life's going to be a good idea.’

P14 (community nurse, 5–10 years exp)

### 4.2 Lack of continuity in promoting prevention

Another perceived challenge was around ongoing care and support for people to self‐manage their lower limb health once the acute cellulitis had been treated. Nurses expressed frustration and concern about perceived lack of continuity of care from care staff and lack of recognition of the need for ongoing care from NHS managers, resulting in their prevention efforts being made in vain. Participants highlighted multiple barriers to involving paid carers in supporting lower limb care such as carer's lack of skills and confidence in applying moisturising creams and compression garments and/or the lack of time to support people with these preventative measures.

[Paid] Carers often say that they're not allowed…Carers not trained to understand the importance of simple skincare and how much of an impact that could have on reducing the likeliness of cellulitis and other skin issues.

P19 (community nurse, >10 years exp)

Another common view was that there is a gap in ongoing support beyond acute care for lower limb care such as skin care and compression due to lack of paid care services and no availability of paid social care services for supporting lower limb care alone (i.e. for people who can self‐care but require extra support with applying moisturising creams and compression garments).

I've got two gentlemen on our caseload who come into us for leg care because they don't fit the criteria for having social services paid carers. They're too independent at the end of the day, that's the problem, but actually, they can't get their stockings on themselves. They don't want to pay for care, for someone to just come round to put a pair of socks on or to put a pair of compression stockings on. We do have that as a bit of an issue.

P2 (practice nurse, 17 years exp)

Nurses discussed how involving a person's support network in the management of their lower limb health could be an important way to address the perceived support gap.

I saw a lady yesterday and she is completely wheelchair bound but she has a son that lives with her, and her legs were amazing. You can tell that they're well hydrated and that they're looked after. It's made a massive difference. She's had problems with her feet, but her skin looks amazing considering that she was 92 so it shows that it can be done…I think it's just having a very supportive son that lives with her and equally she has carers that go in regularly, so she's obviously well looked after.

P4 (practice nurse, 10 years exp)

### 4.3 Practical barriers around compression

In additional to general resource barriers, participants also spoke about the specific resource barriers involved in prescribing compression therapy in terms of lack of access to diagnostic equipment, the challenges of managing competing priorities and navigating complex bureaucratic time‐consuming systems. Participants described specific barriers around lack of access to Doppler machines to assess people's suitability for compression, limited nursing staff capacity to complete the time‐intensive assessment processes as well as ordering/supply issues *for example*, finding the forms difficult; the pharmacy not always having the right product.

‘Probably the biggest one is time constraints. For example, today I've had a message from one of the nurses saying, “Can you come out and do a Doppler with me, please? I'm not signed off and nobody else in the team is in.” She can do it, she's had the training, but she's not been signed off, so I've asked one of the clinical development leads to go with her. There is always that, often resources. Dopplers are one of the things get moved as not a priority when we're up against it because the demand of the service is so much.’

P18 (community nurse, 4 years exp)

## 5 PSYCHOSOCIAL BARRIERS

### 5.1 Changing people's beliefs about self‐management

Participants acknowledged that it can be difficult to get some people ‘on board’ with self‐managing risk factors for recurrence. They expressed frustration with having to confront perceived lack of motivation for self‐management and the difficulties of trying to engage people in lower limb care but reflected that there was ‘only so much that nurses can do’.

It's very variable between patients really as to whether they're particularly interested. That's probably one of the biggest problem things that people are just not motivated really to look after themselves very well. So, it's finding ways to motivate them really to actually self‐care and look after themselves.

P12 (community nurse, >10 years exp)

Perceived difficulties in encouraging self‐management was seen by some participants to be due to a lack of good routine, and not seeing cellulitis as a significant ongoing problem and/or not believing it's necessary once the acute episode has resolved (not seen as a long‐term).

I think when they've got a lot of other things going on in their lives, their legs aren't really the top of their priorities, are they?

P1 (practice nurse, 5–10 years exp)

### 5.2 Social acceptability of lower limb care

Nurses reported issues of social acceptability of self‐management advice. In particular, some men were uneasy about using moisturisers and wearing ‘stockings’. Nurses offered examples of how they addressed such issues.

I find… men can be very funny about putting on moisturisers, so you do have to explain…if it [skin] dries out, it cracks, and that's when you get the infection in, but if you keep it moist, it can stretch, become supple, then you're less likely to get the broken areas and get the cellulitis again.

P21 (community nurse, >10 years exp)

## 6 PHYSICAL BARRIERS

### 6.1 Being unable to reach lower limb to self‐apply emollients and maintain good hygiene

Another perceived barrier to promoting lower limb care was patients being unable to reach lower legs due to age, reduced mobility and frailty, deteriorating eyesight, being overweight or having comorbidities. Being physically unable to reach lower leg was seen as a common barrier to encouraging people self‐applying emollients and maintaining good lower limb hygiene amongst participants.

I would say probably they might well do it initially and then they'll forget to do it, or then they run out of the cream, or they can't physically get to their legs to do it. It's just either a lack of self‐care or not having someone who can do it for them.

P12 (community nurse, 32 years exp)

### 6.2 Physically unable to elevate legs and/or elevate legs high enough

Another physical barrier was the challenge of being able to elevate legs high enough to achieve any benefit. It was apparent that participants commonly perceived promoting leg elevation as at least as challenging, if not more so, than promoting compression or skin care due to physical barriers.

We always say about elevation, but no one ever does it properly. I don't think anyone ever gets their legs up high enough, or for long enough. We always say, pop a pillow under your feet if you're lying in bed so that your legs are higher than your heart…it's obviously quite hard to do to get them really high if you're living on your own.

P6 (practice nurse, 7 years exp)

### 6.3 Struggle to put on compression garments

A further perceived challenge to promoting use of compression was that people very often struggle to put compression garments on by themselves due to the compressive strength of such garments.

I think compression garments are good but very challenging for a lot of patients just because of the sheer method of putting them on. Obviously, they're tight, they're compression, and actually being able to do that daily or even a couple of times a week can be quite challenging for old people if they've got arthritis or anything like that.

P10 (practice nurse, 26 years exp)

While the challenges of compression were commonly acknowledged, some participants felt that adopting a flexible approach and trying to find what's right for the patient can help to maximise concordance with compression. Exploring different options of compression garments and being realistic about what compression strength is feasible at home, even if it is often at a lower compression strength than what they would usually recommend were felt to be important ways to address the challenges of compression garments.

We use wrap type hosiery quite a lot now. That's a real boom because a lot of people that can't get over their feet, or get hosiery on over their feet, or have family members that are themselves a little bit older and haven't got the strength in their hand to get a compression over someone's foot, the wrap type hosiery works really well.

P17 (community nurse, 30 years exp)

## 7 DISCUSSION

This is the first study to explore community and practice nurses' experiences of supporting people at risk of recurrent lower limb cellulitis. Supporting self‐management to reduce the risk of recurrent lower limb cellulitis was viewed as a core part of their role. However, participants expressed a lack of confidence in some aspects of care (such as identifying fungal foot infection) and frustration around the multiple perceived challenges in other aspects of lower limb care.

Lack of time was perceived as a major barrier, but also a perception that preventative treatments and promoting self‐management of lower limb care were not viewed as a priority by healthcare leaders. Other resource barriers were problems with accessing diagnostic equipment and lack of adequate care services for patients unable to self‐care. Perceived barriers also included psychosocial issues such as the social acceptability of lower limb care and the challenge of changing people's attitudes toward self‐management as well as physical barriers such as patients having difficulty elevating their legs, being unable to reach lower limbs to self‐apply emollients, maintaining good hygiene, and putting on compression garments. Participating nurses thought that an invention/support tool to help people self‐manage risk factors for recurrence was needed but had mixed views about the optimal format and primary use of such a support tool.

This novel study adds to the limited research evidence around experiences of managing recurrent lower limb cellulitis and, more broadly, nurses' experiences of encouraging patient self‐management. As well as highlighting challenges, our study found potential strategies to overcome perceived challenges which could inform the development of materials to support patient self‐management of lower limb care. Study recruitment was impacted by the Covid 19 pandemic resulting in a convenience study sample who were likely to be more motivated to engage in research and may not be representative of the primary care nurse population. It is also possible that nurses' frustrations with the multiple challenges of promoting lower limb care were amplified by the pandemic context, although there was a sense in the data that many difficulties pre‐dated covid.

Our findings are supported by previous qualitative research exploring nurses' experiences of facilitating self‐management of chronic health conditions in primary care.^28^, ^29^ Difficulties in improving patient motivation to self‐manage due to lack of resources and adequate organisational support and the value of involving family, friends or carers in encouraging self‐care appear to be common experiences. Concerns about time resource needed for promoting compression are not unfounded, given that randomised controlled trial evidence for the effectiveness of compression, and adherence to compression, involved support for patients from specialist physiotherapists.^30^

Previous qualitative research has explored the views and experiences of recurrent cellulitis amongst people with cellulitis,^6^, ^31^ people with lymphoedema^32^ and healthcare professionals.^33^ Such research has highlighted that many people who have experienced cellulitis are unaware of the risk of repeat episodes and how to prevent them but are keen to find out about ways to prevent repeat episodes.^6^ Our previous mixed methods study exploring views on non‐antibiotic preventative behaviours found wide support for this approach amongst patients who had experienced cellulitis.^34^

Our findings support the need for an increased emphasis on prevention of recurrent cellulitis and more evidence‐based tools to help healthcare professionals and patients reduce the risk of recurrence. Cellulitis is a major reason for antibiotic prescribing in primary care^35^ and causes significant impacts for patients, including pain, anxiety and social and lifestyle impacts.^36^ Current U.K. guidance on the prevention of recurrent cellulitis focuses primarily on the use of prophylactic antibiotics as this is the only intervention with a good evidence base.^37^ Developing and evaluating non‐antibiotic interventions could therefore result in significant reductions in both acute and prophylactic antibiotic use, which is imperative for addressing the urgent problem of increasing antimicrobial resistance, as well as benefits for patients.

In conclusion, this study highlighted several challenges that nurses face with supporting people to manage risk factors for recurrent lower limb cellulitis in terms of resources available to them, as well as the physical and psychosocial capabilities of patients. Nurses identified potential strategies to overcome these challenges, such as greater emphasis on prevention of future recurrence during acute episodes; provision of resources for patients and support networks (paid and unpaid carers) to reinforce knowledge post‐consultation and develop skills for self‐management, but there is an urgent need for health care managers and commissioners to prioritise preventative lower‐limb care. There is also a need to develop and evaluate support materials to help patients reduce their risk of repeat episodes of cellulitis. If effective, such tools could help to reduce cellulitis recurrence rates and reduce need for repeated courses of antibiotics for this painful condition.

## CONFLICT OF INTEREST STATEMENT

None to declare.

## AUTHOR CONTRIBUTIONS

**Ingrid Muller**: Conceptualization (equal); formal analysis (equal); funding acquisition (equal); investigation (equal); methodology (equal); writing – original draft (equal); writing – review & editing (equal). **Emma Teasdale**: Conceptualization (equal); data curation (equal); formal analysis (equal); funding acquisition (equal); investigation (equal); methodology (equal); writing – original draft (equal); writing – review & editing (equal). **Fiona Cowdell**: Formal analysis (equal); funding acquisition (equal); investigation (equal); methodology (equal); writing – review & editing (equal). **Peter Smart**: Funding acquisition (equal); investigation (equal); writing – review & editing (equal). **Miriam Santer**: Conceptualization (equal); formal analysis (equal); funding acquisition (equal); investigation (equal); methodology (equal); project administration (equal); writing – review & editing (equal). **Nick Francis**: Conceptualization (equal); formal analysis (equal); funding acquisition (equal); investigation (equal); methodology (equal); project administration (equal); writing – review & editing (equal).

## ETHICS STATEMENT

Not applicable.

## Data Availability

The data underlying this article will be shared on reasonable request to the corresponding author.
